# Epigenetics of early-life adversity in youth: cross-sectional and longitudinal associations

**DOI:** 10.1186/s13148-022-01269-9

**Published:** 2022-04-08

**Authors:** Jennifer A. Sumner, Simone Gambazza, Xu Gao, Andrea A. Baccarelli, Monica Uddin, Katie A. McLaughlin

**Affiliations:** 1 Department of Psychology, University of California, Los Angeles, Psychology Building 1285, Box 951563, Los Angeles, CA 90095-1563 USA; 2 Department of Clinical Sciences and Community Health, University of Milan, Milan, Italy; 3 Healthcare Professions Department, Fondazione IRCCS Ca’ Granda Ospedale Maggiore Policlinico, Milan, Italy; 4 Department of Environmental Health Sciences, Columbia Mailman School of Public Health, New York, NY USA; 5 Department of Occupational and Environmental Health Sciences, Peking University, Beijing, China; 6 Genomics Program, College of Public Health, University of South Florida, Tampa, FL USA; 7 Department of Psychology, Harvard University, Cambridge, MA USA

**Keywords:** Threat, Deprivation, Abuse, Neglect, DNA methylation

## Abstract

**Background:**

Altered DNA methylation (DNAm) may be one pathway through which early-life adversity (ELA) contributes to adverse mental and physical health outcomes. This study investigated whether the presence versus absence of ELA experiences reflecting the dimensions of threat and deprivation were associated with epigenome-wide DNAm cross-sectionally and longitudinally in a community-based sample of children and adolescents.

**Methods:**

In 113 youths aged 8–16 years with wide variability in ELA, we examined associations of abuse (physical, sexual, emotional; indicating threat-related experiences) and neglect (emotional, physical; indicating deprivation-related experiences) with DNAm assessed with the Illumina EPIC BeadChip array, with DNA derived from saliva. In cross-sectional epigenome-wide analyses, we investigated associations of lifetime abuse and neglect with DNAm at baseline. In longitudinal epigenome-wide analyses, we examined whether experiencing abuse and neglect over an approximately 2-year follow-up were each associated with change in DNAm from baseline to follow-up.

**Results:**

In cross-sectional analyses adjusting for lifetime experience of neglect, lifetime experience of abuse was associated with DNAm for four cytosine-phosphodiester-guanine (CpG) sites (cg20241299: coefficient = 0.023, *SE* = 0.004; cg08671764: coefficient = 0.018, *SE* = 0.003; cg27152686: coefficient = − 0.069, *SE* = 0.012; cg24241897: coefficient = − 0.003, *SE* = 0.001; FDR < .05). In longitudinal analyses, experiencing neglect over follow-up was associated with an increase in DNAm for one CpG site, adjusting for abuse over follow-up (cg03135983: coefficient = 0.036, *SE* = 0.006; FDR < .05).

**Conclusions:**

In this study, we identified examples of epigenetic patterns associated with ELA experiences of threat and deprivation that were already observable in youth. We provide novel evidence for change in DNAm over time in relation to ongoing adversity and that experiences reflecting distinct ELA dimensions may be characterized by unique epigenetic patterns.

**Supplementary Information:**

The online version contains supplementary material available at 10.1186/s13148-022-01269-9.

## Introduction

Early-life adversity (ELA) is linked to deleterious mental and physical health outcomes over the lifespan [1, 2]. ELA refers to experiences that represent a deviation from the expectable environment and require adaptation, encompassing experiences such as physical, sexual, and emotional abuse, neglect, and institutional rearing [3]. The epigenome, including DNA methylation (DNAm), influences whether genes are expressed, and provides a molecular context for how the genome is influenced by environmental experience. DNAm may be a pathway by which ELA—a potent environmental exposure—becomes biologically embedded and contributes to adverse mental and physical health [4].

Growing work has explored whether ELA is associated with differential DNAm patterns (for a review, see [5]). Most research has employed a candidate gene approach, focusing on probes in genes related to the hypothalamic–pituitary–adrenal (HPA) axis and stress-related neurotransmitter genes. However, candidate gene studies are limited by the available understanding of the neurobiology associated with ELA to inform gene selection. Accordingly, more recent work has employed hypothesis-free epigenome-wide approaches. Some [6–12], but not all [13, 14], epigenome-wide association studies (EWAS) suggest ELA is associated with differential DNAm patterns. Many studies measured retrospectively reported ELA and DNAm in adulthood, with fewer EWAS in youths. However, a recent meta-analysis documented meaningful concerns with using retrospective reports of ELA in adults, showing poor concordance with prospective measures of ELA assessed in childhood [15]. Additionally, in retrospective studies, it is unclear whether DNAm differences emerged after ELA or if they reflect later experiences. For example, ELA is associated with toxins such as tobacco [16], which has documented epigenetic markers [17] and has been shown to confound some ELA-DNAm associations [13]. Natural variation in DNAm as a result of aging over the lifespan [18] can also make it challenging to extend findings from adult samples to youths. Conducting work in youths provides an opportunity to examine epigenetic patterns that may be observed relatively soon after experiencing ELA.

Furthermore, much epigenetics research has aggregated all forms of ELA into a single exposure (present vs. absent) rather than considering particular ELA types. Accumulating evidence suggests distinct effects of ELA involving the dimensions of threat (experiences that reflect potential physical harm, such as abuse and other violence) and deprivation (involving the absence of expected environmental inputs, such as neglect) on neurobiological development [19–21]. To date, three EWAS in younger individuals considered particular ELA types. In the first, Cecil et al. [6] documented unique and shared associations of ELA types with DNAm when examining differentially methylated probes linked to physical abuse, sexual abuse, and physical neglect in a relatively small sample. In two large population-based cohorts, Marzi et al. [13] and Dunn et al. [22] examined associations between numerous ELA types in childhood and/or adolescence (e.g., sexual abuse, physical abuse, neglect, peer victimization) and DNAm. However, few robust associations emerged. In these EWAS, ELA types were treated as distinct exposures with potentially unique mechanisms (i.e., a specificity approach) rather than considered as experiences that might share core features, such as threat and deprivation (i.e., a dimensional approach). Thus, despite growing evidence suggesting that studying key dimensions of environmental experience occurring in multiple ELA types can shed light on unique neurodevelopmental mechanisms [20], DNAm research employing this approach is lacking.

Finally, nearly all epigenome-wide research on ELA and DNAm has been cross-sectional. Even in longitudinal studies, DNAm has typically been measured only once [13, 22]. In one exception, Martins et al. [23] found that child maltreatment (aggregating across experiences of abuse and neglect) and greater adversity (reflecting a more global stress burden) were associated with more blunted changes in DNAm over approximately 2 years during early childhood. Further research is needed to examine how the epigenome changes during childhood and adolescence after ELA.

In this longitudinal study, we investigated whether the presence versus absence of ELA experiences reflecting the dimensions of threat (i.e., abuse) and deprivation (i.e., neglect) were associated with DNAm in a community-based sample of 113 youths aged 8–16 years at baseline. First, we examined whether lifetime abuse and neglect, as well as the frequency and severity of those experiences, were each associated with DNAm measured at baseline in epigenome-wide analyses. Second, we investigated whether experiencing abuse or neglect over an approximately 2-year follow-up period was related to epigenome-wide changes in DNAm from baseline to follow-up. In secondary analyses, we conducted hypothesis-driven cross-sectional and longitudinal analyses of DNAm in candidate genes implicated in the stress response.

## Methods

### Participants and procedure

Youths aged 8–16 years and a caregiver were recruited from the community to participate in a study examining ELA, emotion regulation, and psychopathology (see Additional file 1: Methods). ELA was queried at baseline and follow-up approximately 2 years later, and youths provided saliva samples for DNAm at both assessments. Study procedures were approved by the University of Washington Institutional Review Board. Caregivers provided written informed consent; youths provided written assent. Of the 262 youths enrolled in the parent study [24], a total of 161 participants were included in a sub-sample that provided neuroimaging data and saliva samples for epigenetic analysis [25]. Of those 161 participants, 113 (70.2%) provided saliva samples at both assessments and had DNAm levels assayed for the current analyses. These 113 participants comprised the analytic sample.

### ELA

ELA was assessed using a multi-informant, multi-method approach (see Additional file 1: Methods). At baseline, youths completed interviews and questionnaires assessing lifetime maltreatment experiences (e.g., physical, sexual, and emotional abuse, emotional and physical neglect) and violence exposure, including the Childhood Experiences of Care and Abuse interview [26], the Violence Exposure Scale for Children-Revised [27], the Childhood Trauma Questionnaire [28], and the UCLA Posttraumatic Stress Disorder (PTSD) Reaction Index [29]. Caregivers completed questionnaires assessing youths’ lifetime experiences of abuse, violence exposure, and other adversities, including the Conflict Tactics Scale-Parent Child Version [30], the Juvenile Victimization Questionnaire lifetime caregiver report [31], the caregiver version of the UCLA PTSD Reaction Index [29], the short form of the U.S. Department of Agriculture’s Food Security Scale [32], and the Home Observation for Measurement of the Environment-Short Form [33]. At follow-up, youths and caregivers completed these interview and/or questionnaire measures with respect to ELA experiences that occurred to youths between baseline and follow-up.

Across these validated ELA measures, multiple experiences reflecting threat and deprivation were assessed. We combined youth and caregiver baseline reports using an “or” rule to indicate presence vs. absence of lifetime abuse (physical, sexual, and/or emotional abuse; indicating threat-related experiences). The presence versus absence of lifetime neglect (emotional and/or physical neglect; indicating deprivation-related experiences) was based on youth report. Follow-up reports were used to indicate whether abuse or neglect occurred over follow-up. Although we focused primarily on whether youths underwent experiences characterized by threat or deprivation (i.e., presence vs. absence of abuse or neglect) when examining differences in DNAm, we also investigated continuous threat and deprivation composites. These composites reflect the frequency and severity of ELA experiences and were calculated by summing the number of threat and deprivation experiences, respectively, endorsed by youth and/or caregiver (see Additional file 1: Methods).

### DNAm

Saliva samples were collected at baseline and follow-up using Oragene® kits. DNA extraction and bisulfite conversion using the EZ-96 DNA Methylation kit were conducted by AKESOgen. Methylation of > 850,000 cytosine-phosphodiester-guanine (CpG) sites was measured using the Illumina EPIC BeadChip array. To reduce within-participant variability, baseline and follow-up samples for a participant were assayed simultaneously on the same chip using a balanced chip design.

DNAm data cleaning and pre-processing were conducted using the minfi R package [34]. CpGs with detection *p* values > 0.01 in > 5% of individuals were removed. Cross-hybridizing, genetically confounded, and sex chromosome probes were removed. Data cleaning excluded 125,666 probes after quality control. DNAm data were pre-processed using the Illumina-type background correction, dye-bias adjustment, and normal-exponential out-of-band normalization, which were used to generate methylation status. The methylation status of a CpG site was quantified as a β-value from 0–1 (no methylation to full methylation).

### Covariates

Analyses adjusted for age and sex. As poverty is a context that can increase the likelihood of experiencing ELA and other environmental risks that can impact DNAm [35], we adjusted for family income-to-needs ratio. Caregivers reported household income at baseline and follow-up; income-to-needs ratio was calculated by dividing household income by the US census-defined poverty line for their family size. To account for differences in cell type proportions across samples [36], we generated cell-type principal components (PCs) using the RefFreeEWAS R package [37]. Although race/ethnicity was self-reported, we used the first five ancestry PCs, derived from genetic data collected for a separate investigation, to account for population stratification (confounding due to genetic ancestry) [38]. As tobacco use is a potential confounder of ELA-DNAm associations [13], youth tobacco use—reported by youths or caregivers on the Youth Self Report or Child Behavior Checklist [39] at baseline and follow-up—was covaried in sensitivity analyses.

### Analytic approach

For our first aim, we investigated cross-sectional associations of lifetime abuse and neglect (presence vs. absence) at baseline with DNAm of 740,889 CpG sites using linear mixed effects models in the CpGassoc R package [40]. Models adjusted for age, sex, income-to-needs ratio, the first five cell-type PCs and ancestry PCs, and random batch effects of DNAm measurement. For our second aim, we tested associations of abuse and neglect (presence vs. absence) over follow-up with change in 737,826 CpG sites from baseline to follow-up (3063 CpGs did not survive pre-processing for follow-up DNAm). For these longitudinal analyses, we used generalized linear models in the ewaff R package (https://github.com/perishky/ewaff), adjusting for covariates as in cross-sectional analyses (with income-to-needs ratio based on averaging baseline and follow-up reports and including both cell-type PCs from baseline and follow-up as covariates). Variations in CpG sites were estimated with the difference in adjusted *β*-values between baseline and follow-up (*β*_follow-up _– *β*_baseline_), which accounted for batch effects using the ComBat R package [41]. We also conducted cross-sectional and longitudinal EWAS with the continuous threat and deprivation composites.

In secondary analyses, we interpreted results for CpG sites within nine candidate genes implicated in the HPA axis and stress-related neurodevelopmental and neurotransmitter pathways: *NR3C1* (glucocorticoid receptor gene) [42], *FKBP5* (FK506 binding protein 5 gene) [43], *CRHR1* (corticotropin-releasing hormone receptor gene) [44], *AVP* (a gene encoding vasopressin) [45], *SLC6A4* (serotonin transporter gene) [46], *HTR3A* (a gene encoding a serotonin receptor) [47], *MAOA* (monoamine oxidase A gene) [48], *BDNF* (brain-derived neurotrophic factor gene) [49], and *OXTR* (oxytocin receptor gene) [50]. CpG sites in each gene were identified by searching the EPIC annotation library for (1) CpGs within the positions of the genes (derived from GRCh37/hg19 UCSC Genome Browser) and (2) gene names.

In the analyses described above, we first examined each ELA type independently. We then estimated models with both ELA types to evaluate unique associations given high co-occurrence of threat- and deprivation-related experiences. For example, at baseline, lifetime abuse and neglect were significantly positively correlated (phi = 0.50, *p* < 0.0001), as were the threat and deprivation composites (*r* = 0.68, *p* < 0.0001). The model including both ELA types represents the most conservative test, as it removes variance associated with one ELA type from the analysis examining the relation of the other ELA type with DNAm [20]. Further, this approach allowed us to consider the unique associations of particular types of ELA experiences. Additionally, sensitivity analyses covaried youth tobacco use to examine the robustness of significant findings in the main analyses. In sum, a total of nine cross-sectional and nine longitudinal EWAS were conducted. Multiple testing was accounted for by controlling the false discovery rate (FDR) at 5%. For candidate gene analyses, the FDR multiple correction was based on the total number of associations tested across genes.

Additionally, secondary gene ontology (GO) analyses explored the biological function shared by genes corresponding to CpG sites identified in EWAS. We selected CpG sites uniquely associated with (1) abuse and (2) neglect (adjusting for covariates and the other ELA type) in both cross-sectional and longitudinal analyses prior to FDR-correction. Given the exploratory and hypothesis-generating nature of the GO analyses, we selected CpG sites based on nominal significance. We conducted analyses using the *gometh* function in the missMethyl R package [51].

## Results

### Sample characteristics

The analytic sample was 42.5% female and diverse with respect to race/ethnicity and income-to-needs ratio, with a mean age of 12.2 years at baseline (Table 1). At baseline, 52.2% of participants experienced abuse during their lifetime, 26.5% experienced neglect, and 24.8% (*n* = 28) experienced both abuse and neglect. Mean follow-up time was 1.7 years. Over follow-up, 31.0% of participants experienced abuse; 11.5% experienced neglect. Reported tobacco use was low at baseline and follow-up.

**Table 1:** Participant characteristics for the analytic sample (*N* = 113)

Characteristic	*M* (*SD*) or % (*n*)	Range	*n*
Age at baseline, years	12.2 (2.5)	8.0–17.0	113
Female sex	42.5 (48)		113
Race/ethnicity			113
White	44.2 (50)		
Black	21.2 (24)		
Latino	11.5 (13)		
Asian	10.6 (12)		
Other	12.4 (14)		
Family income-to-needs ratio, baseline	3.9 (2.8)	0.1–10.4	102
Family income-to-needs ratio, follow-up	3.7 (2.8)	0.1–10.4	107
Time between baseline and follow-up, years	1.7 (0.6)	0.8–3.2	113
Tobacco use, baseline	0.9 (1)		112
Tobacco use, follow-up	7.2 (8)		111
*Early-life adversity*			
Lifetime experience of abuse, baseline	52.2 (59)		113
Lifetime experience of neglect, baseline	26.5 (30)		113
Lifetime threat composite, baseline	4.9 (3.7)	0–14	113
Lifetime deprivation composite, baseline	0.9 (1.1)	0–4	113
Experience of abuse during follow-up	31.0 (35)		113
Experience of neglect during follow-up	11.5 (13)		113
Threat composite during follow-up	2.6 (2.7)	0–12	113
Deprivation composite during follow-up	0.9 (1.1)	0–4	113

*M* mean, *SD* standard deviation

Descriptive statistics for participants in the analytic sample were very similar to those for the larger subset of cohort participants who were eligible to provide saliva samples for epigenetic analyses (Additional file 1: Table S1).

### Cross-sectional analyses

**Fig. 1:**
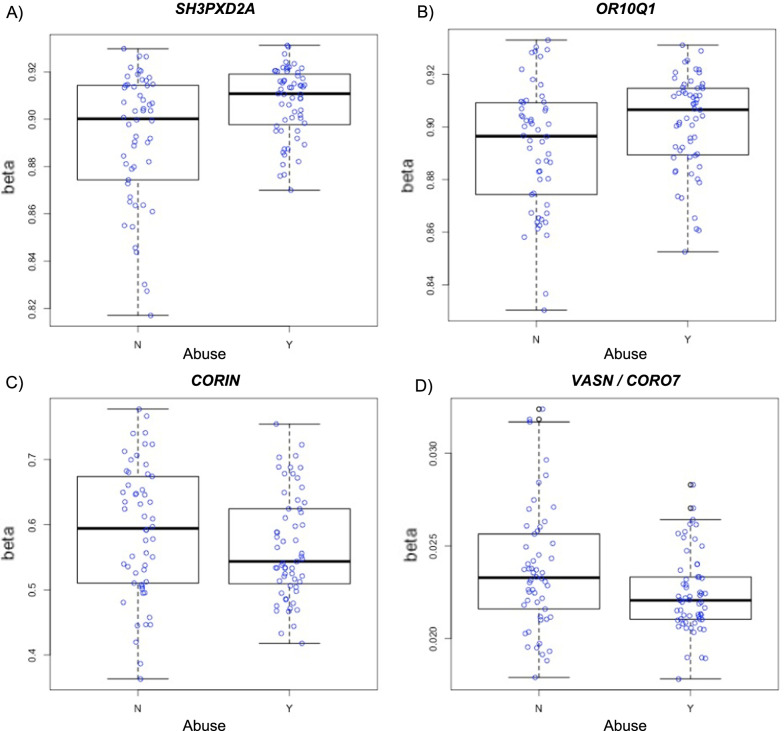
Box plots of DNA methylation *β*-values for participants with and without lifetime abuse for four CpG sites uniquely associated with lifetime abuse in cross-sectional analyses, adjusting for lifetime neglect

In the cross-sectional EWAS, 15 CpG sites were significantly associated with lifetime abuse (Table 2). When further adjusting for lifetime neglect, one CpG site annotated to the *OR10Q1* gene (cg08671764) remained significantly associated with abuse, and three other CpG sites were identified as being uniquely associated with lifetime abuse. Figure 1 presents box plots of DNAm *β*-values for participants with and without lifetime abuse for these sites. Not only were these four CpG sites associated with lifetime abuse versus neglect based on statistical significance, but the effect estimates for these sites in the cross-sectional EWAS with both ELA types in the model were in opposite directions for abuse and neglect, further suggesting distinct relations (Additional file 1: Table S2). One CpG site was significantly associated with lifetime neglect, but not when adjusting for lifetime abuse (Table 2). The DNAm β-values for the CpG sites significantly associated with lifetime abuse or neglect in the cross-sectional EWAS were similar in males and females (Additional file 1: Fig. S1). No significant associations emerged for continuous lifetime threat and deprivation composites. We investigated the concordance of findings for the dichotomous ELA type variables and the continuous ELA composite variables by examining the estimates for the threat and deprivation composites for CpG sites that were significantly associated with either lifetime abuse or neglect, respectively, in the cross-sectional EWAS. Although estimates for (1) abuse and threat and (2) neglect and deprivation were in the same direction, the coefficients for the continuous composite variables were smaller than for the dichotomous variables (Additional file 1: Table S3).

**Table 2:** Probes significantly associated with lifetime experience of abuse and neglect at baseline in cross-sectional epigenome-wide analyses

Probe ID	Position	Gene	Gene region feature^a^	Coefficient (SE)	FDR^b^
*Lifetime experience of abuse*					
Model 1^c^					
cg08671764	chr11:57996369	*OR10Q1*	1st exon	0.016 (0.003)	.018
cg19454603	chr11:107730446	*SLC35F2*	TSS1500	− 0.005 (0.001)	.020
cg05462437	chr14:70528945	*SLC8A3*	5’UTR	0.022 (0.004)	.020
cg25625296	chr1:1145848	Unassigned	Unassigned	− 0.029 (0.005)	.020
cg18348616	chr10:97470256	*ENTPD1*	TSS1500	− 0.101 (0.018)	.027
cg26357241	chr1:109289876	*STXBP3*	Body	− 0.012 (0.002)	.034
cg06383709	chr16:26148977	*HS3ST4*	3’UTR	0.016 (0.003)	.034
cg00992846	chr12:122906318	*CLIP1*	5’UTR	− 0.003 (0.000)	.034
cg16793662	chr2:55407554	*CLHC1*	Body	− 0.065 (0.012)	.036
cg07451097	chr4:143641250	*INPP4B*	5’UTR	0.046 (0.008)	.036
cg10091662	chr10:22609897	*BMI1*	TSS200	− 0.017 (0.003)	.047
cg15350036	chr7:86973677	*TP53TG1*	TSS1500	− 0.054 (0.010)	.047
cg03727700	chr10:115614022	*DCLRE1A*	TSS1500	− 0.004 (0.001)	.047
cg02047211	chr17:78932697	*RPTOR*	Body	0.011 (0.002)	.047
cg04866720	chr2:150588473	*LOC101929231*	Body	0.009 (0.002)	.047
Model 2^d^					
cg20241299	chr10:105362799	*SH3PXD2A*	Body	0.023 (0.004)	.013
cg08671764	chr11:57996369	*OR10Q1*	1st exon	0.018 (0.003)	.013
cg27152686	chr4:47645625	*CORIN*	Body	− 0.069 (0.012)	.019
cg24241897	chr16:4421892	*VASN*/*CORO7*	1st exon	− 0.003 (0.001)	.023
*Lifetime experience of neglect*					
Model 1^c^					
cg00284420	chr16:87311948	Unassigned	Unassigned	− 0.009 (0.001)	.002

*Chr* chromosome, *FDR* false discovery rate, *SE* standard error

^a^Gene region feature category describing the CpG position from the UCSC Genome Browser. TSS200 = 0–200 bases upstream of the transcriptional start site (TSS); TSS1500 = 200–1500 bases upstream of the TSS; 5'UTR = within the 5' untranslated region (UTR), between the TSS and the ATG start site; Body = between the ATG and stop codon; irrespective of the presence of introns, exons, TSS, or promoters; 3'UTR = between the stop codon and poly A signal

^b^Probes significant at FDR < .05

^c^Model 1 adjusted for age and income-to-needs ratio at baseline, sex, first five cell-type principal components, first five ancestry principal components, and random batch effects of DNA methylation measurement

^d^Model 2 adjusted for Model 1 covariates and lifetime neglect

In sensitivity analyses adjusting for tobacco use, 14 of the 15 CpG sites associated with lifetime abuse remained significant, and 21 additional CpG sites were significantly related to lifetime abuse (Additional file 1: Table S4). All four CpG sites uniquely associated with lifetime abuse remained significant in these sensitivity analyses, and four additional sites were identified. The CpG site significantly associated with lifetime neglect (although not when adjusting for abuse) remained linked to neglect when adjusting for tobacco (Additional file 1: Table S4).

Secondary candidate gene analyses identified a few sites significantly associated with lifetime abuse and/or neglect (Table 3). One CpG site in *CRHR1* was associated with abuse (including when adjusting for tobacco), one in *FKBP5* was associated with neglect (including when adjusting for abuse and tobacco), and one in *BDNF* was associated with abuse when adjusting for tobacco. The same site in *OXTR* was associated with lifetime abuse and neglect when adjusting for the other ELA type and tobacco, but in opposite directions for abuse and neglect.

**Table 3:** Probes in candidate genes significantly associated with lifetime experience of abuse and neglect at baseline in cross-sectional analyses

Gene	Probe	Model	Coefficient (SE)	FDR^a^
Lifetime experience of abuse				
*CRHR1*	cg16830379	1^b^	− 0.015 (0.004)	.010
*CRHR1*	cg16830379	3^d^	− 0.015 (0.004)	.013
*OXTR*	cg03710862	2^c^	− 0.020 (0.006)	.013
*OXTR*	cg03710862	4^e^	− 0.019 (0.006)	.018
*BDNF*	cg25928860	3^f^	− 0.031 (0.009)	.043
Lifetime experience of neglect				
*OXTR*	cg03710862	5^**f**^	0.018 (0.005)	.022
*OXTR*	cg03710862	6^g^	0.018 (0.005)	.021
*FKBP5*	cg03098337	1^b^	− 0.009 (0.002)	.014
*FKBP5*	cg03098337	3^d^	− 0.008 (0.002)	.045
*FKBP5*	cg03098337	5^f^	− 0.010 (0.003)	.009
*FKBP5*	cg03098337	6^g^	− 0.010 (0.003)	.022

*Chr* chromosome, *FDR* false discovery rate, *SE* standard error

^a^Probes significant at FDR < .05

^b^Model 1 adjusted for age and income-to-needs ratio at baseline, sex, first five cell-type principal components, first five ancestry principal components, and random batch effects of DNA methylation measurement

^c^Model 2 adjusted for Model 1 covariates and lifetime neglect

^d^Model 3 adjusted for Model 1 covariates and tobacco use

^e^Model 4 adjusted for Model 2 covariates and tobacco use

^f^Model 5 adjusted for Model 1 covariates and lifetime abuse

^g^Model 6 adjusted for Model 5 covariates and tobacco use

### Longitudinal analyses

In the longitudinal EWAS, experiencing neglect over follow-up was associated with an increase in DNAm levels for one CpG site annotated to the *ZFAT* gene (cg03135983; chromosome 8:135724038; gene region feature = body; coefficient = 0.036, *SE* = 0.006, FDR = 0.016). The differences in DNAm *β*-values between baseline and follow-up for this site were similar in males and females (Additional file 1: Fig. S2). Results remained essentially unchanged when further adjusting for abuse over follow-up (coefficient = 0.036, *SE* = 0.006, FDR = 0.022) and tobacco use (coefficient = 0.036, *SE* = 0.006, FDR = 0.049). Moreover, in the longitudinal EWAS with both abuse and neglect in the model, the estimate for cg03135983 for experience of abuse over follow-up was much smaller than the estimate for neglect, suggesting distinct relations for the ELA types (coefficient = 0.000, *SE* = 0.004, FDR = 0.999). Neither abuse over follow-up nor follow-up threat and deprivation composites was significantly associated with DNAm change. For the CpG site significantly associated with experiencing neglect over follow-up (cg031359830), the estimate for the follow-up deprivation composite was in the same direction as for the dichotomous neglect variable but smaller in size (coefficient = 0.006, *SE* = 0.002, FDR = 0.700), similar to what was observed in cross-sectional analyses.

Longitudinal candidate gene analyses identified only one site (in *CRHR1*; cg16830379) that was significantly associated with neglect over follow-up (coefficient = 0.020, *SE* = 0.006, FDR = 0.040). This coefficient remained significant when adjusting for abuse over follow-up (coefficient = 0.020, *SE* = 0.006, FDR = 0.047) but not tobacco.

### Ontology analyses

We found 8238 CpG sites that were associated with abuse (adjusting for neglect) with uncorrected, nominally significant *p* values < 0.05. Secondary GO analyses identified 16 significant pathways after FDR-correction; the top 4 were related to nucleoplasm, nuclear part, nuclear lumen, and nucleus (Additional file 1: Table S5). No significant pathways were associated with neglect.

## Discussion

Epigenetic pathways may provide a molecular mechanism by which ELA translates into differential health outcomes. This is the first study to conduct cross-sectional and longitudinal associations of ELA with DNAm from an epigenome-wide perspective in children and adolescents. Furthermore, we defined ELA based on dimensional models of early experience [20], focusing on experiences of abuse (reflecting the dimension of threat) and neglect (reflecting the dimension of deprivation). A number of genome-wide significant findings emerged, indicating that experiences reflecting threat and deprivation were characterized by different DNAm patterns. Lifetime abuse was associated with DNAm for four CpG sites in cross-sectional analyses when also adjusting for lifetime neglect. Additionally, neglect over follow-up was associated with change in DNAm for one CpG site, adjusting for abuse over follow-up. Moreover, models that mutually adjusted for abuse and neglect revealed substantially different associations for these types of ELA. These findings suggest that experiences across various dimensions of ELA may be characterized by distinct epigenetic patterns already observable in youth.

In cross-sectional analyses, lifetime abuse emerged as the ELA experience most associated with differential DNAm. Numerous genome-wide significant CpG sites were identified in initial comparisons of youths with and without lifetime abuse. Additionally, four sites distinguished youths with and without abuse when further adjusting for lifetime neglect, suggesting differences in DNAm that might be unique to experiences of abuse that do not overlap with experiences of neglect. Specifically, lifetime experience of abuse was associated with higher DNAm levels of sites annotated to the SH3 and PX Domains 2 (*SH3PXD2A*) and olfactory receptor family 10 subfamily Q member 1 (*OR10Q1*) genes, and with lower DNAm levels of sites annotated to the CORIN (*CORIN*) and vasorin (*VASN*)/coronin 7 (*CORO7*) genes (cg24241897 was annotated to a region near *VASN* and *CORO7*). *SH3PXD2A* encodes a scaffolding protein (Tks5) involved in the production and regulation of invadopodia and podosomes, which influence cellular migration and invasion [52]. Furthermore, Tks5-related invadopodia activity has been implicated in tumor growth and metastasis [53], and Tks5 has been linked to cancer [54]. With respect to ELA, differential DNAm of a CpG site annotated to *SH3PXD2A* was found in former indentured laborers exposed to physical, emotional, and sexual abuse as children compared to controls [9]. Although this CpG site (cg11014810) was different than what we identified, it was annotated to the gene body, as was the site we identified. Further, the consistent finding of ELA with methylation of this gene is noteworthy, particularly as the experiences of the former indentured laborers aligned with our abuse definition.

The other genes implicated in cross-sectional EWAS of lifetime abuse have been linked to ELA-related phenotypes and/or have biological functions with relevance to physical health consequences of ELA. For example, *OR10Q1* is a protein-coding gene in the olfactory receptor gene family [55]. Research in a small sample of trauma-exposed adults found differences in olfactory receptor-related gene expression in individuals with and without PTSD, the quintessential trauma-related mental disorder [56]. The *CORIN* and *VASN*/*CORO7* genes may have relevance for adverse physical health consequences associated with ELA experiences of abuse [1]. Corin, or atrial natriuretic peptide (ANP)-converting enzyme, is encoded by the *CORIN* gene and adapts ANP—a cardiac hormone that regulates blood pressure—into biologically active components [57]. *VASN* codes for a type 1 transmembrane glycoprotein, vasorin [58]. Vasorin regulates vascular repair in response to injury, inhibits signaling of transforming growth factor-beta, and may play a role in tumor formation [58, 59]. Additionally, lower DNAm at a CpG site near *CORO7* has been associated with obesity in youths [60]. *CORO7* encodes a protein involved in Golgi complex structure and maintenance and regulation of energy homeostasis [60]. Altogether, it is possible that these epigenetic patterns reflect mechanisms contributing to risk for cancer, cardiovascular disease, and obesity among children who have experienced abuse and other forms of violence, although this remains to be examined.

Only one significant finding emerged in the longitudinal EWAS. Specifically, neglect over follow-up was associated with greater increases in DNAm in a CpG site annotated to the zinc finger and AT-hook domain containing (*ZFAT*) gene. *ZFAT* is a protein-coding gene associated with vulnerability for autoimmune thyroid disease, and overexpression is linked to down-regulation of genes involved in the immune response [61]. ELA, including neglect, has been associated with immune system dysregulation and autoimmune conditions [62, 63]. Furthermore, though different than the site detected in the current study, sites in zinc finger protein-related genes have been linked to ELA [23] and PTSD in EWAS [64, 65]. Given the significant longitudinal finding for this CpG site, we explored whether it showed particular patterns of variation from birth through late adolescence using a recently developed online DNAm trajectory mapping resource from the Epidelta Project [66]. In the Epidelta Project results, there was no evidence of significant change over the first 18 years of life for this CpG site based on the Bonferroni-significant *p*-value threshold. However, the Epidelta Project examined longitudinal trajectories of DNAm levels derived from cord blood and peripheral blood samples, whereas we examined change in DNAm levels derived from saliva samples. Given the tissue-specific nature of DNAm, it is possible that modeling longitudinal trajectories of DNAm using saliva samples could generate different results. Future research is needed to better understand patterns of change over development in childhood and adolescence for this site annotated to the *ZFAT* gene. Furthermore, the overall relative lack of significant findings with respect to change in DNAm over time is consistent with the one other study of ELA and change in epigenome-wide DNAm in youths, which observed substantial stability in overall DNAm patterns over approximately 2 years during early childhood [23]. Thus, it is possible that a longer period of follow-up is needed to observe more robust changes in DNAm as a result of adverse experiences in childhood and adolescence.

Few significant findings emerged in secondary candidate gene analyses—the approach employed in most research on ELA and epigenetics in youths—or when considering continuous threat and deprivation composites. The lack of consistent results parallels prior research [13]. Furthermore, none of the candidate gene sites emerged in EWAS, and none of the sites identified in EWAS were tied to physiological systems examined in candidate gene research. Additionally, though based on uncorrected, nominally significant *p*-values, exploratory GO analyses suggested that pathways related to nucleus development may be particularly associated with abuse. Further research is needed to validate this preliminary finding, and it may indicate a future direction for examining the impact of this type of ELA on biological formation or modification of the nucleus. With regard to the threat and deprivation composites, it is likely that a one-unit change in these continuous metrics of the frequency and severity of ELA experiences was not potent enough to produce changes in epigenome-wide DNAm. Dichotomous measures capturing the presence versus absence of threat- and deprivation-related experiences may be more powerful for detecting these associations.

Several limitations merit acknowledgement. First, the sample size was small, and replication of findings is needed. Further, because only participants from the parent study who provided saliva samples were able to be included in this study of DNAm, selection bias is a potential concern. However, the response rate of those eligible to participate (70.2%) was good, and the analytic sample did not differ meaningfully in sociodemographic composition from the total sample. Due to the small sample size, we also had limited statistical power to test for sub-group differences in associations of ELA with DNAm, such as differences by sex. However, for CpG sites identified in cross-sectional and longitudinal analyses, we demonstrated that the DNAm *β*-values were similar in males and females. Our findings appear to reflect DNAm differences associated with ELA experiences that may be present in both males and females and thus could be generalizable to mixed-sex samples of youths. Well-powered EWAS with large sample sizes are needed to determine whether sex-specific associations are also present. Additionally, although we describe some biological processes associated with genes corresponding to significant CpG sites, functional analyses are needed to understand whether DNAm findings have consequences for gene expression and beyond. Second, DNAm was assessed from saliva. Given the tissue-specific nature of DNAm, use of peripheral samples has limits when drawing conclusions to brain-related processes that could result from ELA (e.g., psychiatric disorders). Third, analyses were limited to EPIC array sites. We also focused on DNAm levels at individual CpG sites, and future research considering other epigenetic markers of ELA (e.g., DNAm age) is warranted. Fourth, in the current investigation, we were unable to examine directly whether genetic effects influenced DNAm levels. Not only is DNAm impacted by genetic variation [67], but recent work has reported significant gene-environment correlations for childhood maltreatment [68] and suggests that considering Gene x ELA interactions may help explain interindividual variability in DNAm over the life course [69]. To explore whether the CpG sites we identified as uniquely associated with abuse or neglect in our EWAS might be influenced by genetic variation, we searched the GoDMC Database [70]. Only one of the CpG sites was associated with known methylation quantitative trait loci, specifically the CpG site associated with neglect over follow-up in longitudinal analyses. Although these findings suggest that genetic variation was unlikely to substantially affect the majority of our results, future ELA research integrating genetic and epigenetic data are needed. Despite these limitations, our study is characterized by several strengths that make our investigation unique. We used a multi-method, multi-informant approach to assessing ELA from a dimensional framework, addressed cross-sectional and longitudinal associations of ELA with DNAm, and adjusted for important confounders, including tobacco use, which accounted for associations of ELA with DNAm in prior research [13].

## Conclusions

We found that ELA experiences are associated with several epigenetic markers that can already be detected in youth. Although we did not detect a large number of genome-wide-significant effects, distinct results were observed for experiences characterized by threat versus deprivation, suggesting that considering dimensional frameworks when examining the consequences of ELA—rather than a “one-size-fits-all” approach—holds promise. Given that ELA-epigenetic associations have been detected over the life course, research needs to examine whether epigenetic patterns linked to experiences of abuse and neglect in youth persist or change over the lifespan. Further research will also be needed to delineate whether the epigenetic findings identified have consequences for mental and physical health in youth and beyond.

## Supplementary Information

**Additional file 1**. Supplemental Methods, Tables, and Figures.

## Data Availability

The datasets used and/or analyzed during the current study are available from the corresponding author on reasonable request.

## References

[1] Felitti VJ, Anda RF, Nordenberg D, Williamson DF, Spitz AM, Edwards V, Koss MP, Marks JS (1998). Relationship of childhood abuse and household dysfunction to many of the leading causes of death in adults: the Adverse Childhood Experiences (ACE) Study. Am J Prev Med.

[2] McLaughlin KA, Green JG, Gruber MJ, Sampson NA, Zaslavsky AM, Kessler RC (2012). Childhood adversities and first onset of psychiatric disorders in a national sample of US adolescents. Arch Gen Psychiatry.

[3] McLaughlin KA (2016). Future directions in childhood adversity and youth psychopathology. J Clin Child Adolesc Psychol.

[4] Jaenisch R, Bird A (2003). Epigenetic regulation of gene expression: how the genome integrates intrinsic and environmental signals. Nat Genet.

[5] Cecil CA, Zhang Y, Nolte T (2020). Childhood maltreatment and DNA methylation: a systematic review. Neurosci Biobehav Rev.

[6] Cecil CA, Smith RG, Walton E, Mill J, McCrory EJ, Viding E (2016). Epigenetic signatures of childhood abuse and neglect: implications for psychiatric vulnerability. J Psychiatr Res.

[7] Cicchetti D, Hetzel S, Rogosch FA, Handley ED, Toth SL (2016). An investigation of child maltreatment and epigenetic mechanisms of mental and physical health risk. Dev Psychopathol.

[8] Houtepen LC, Hardy R, Maddock J, Kuh D, Anderson EL, Relton CL, Suderman MJ, Howe LD (2018). Childhood adversity and DNA methylation in two population-based cohorts. Transl Psychiatry.

[9] Marinova Z, Maercker A, Küffer A, Robinson MD, Wojdacz TK, Walitza S, Grünblatt E, Burri A (2017). DNA methylation profiles of elderly individuals subjected to indentured childhood labor and trauma. BMC Med Genet.

[10] Naumova OY, Lee M, Koposov R, Szyf M, Dozier M, Grigorenko EL (2012). Differential patterns of whole-genome DNA methylation in institutionalized children and children raised by their biological parents. Dev Psychopathol.

[11] Roberts AL, Gladish N, Gatev E, Jones MJ, Chen Y, MacIsaac JL, Tworoger SS, Austin SB, Tanrikut C, Chavarro JE, Baccarelli AA, Kobor MS (2018). Exposure to childhood abuse is associated with human sperm DNA methylation. Transl Psychiatry.

[12] Yang B-Z, Zhang H, Ge W, Weder N, Douglas-Palumberi H, Perepletchikova F, Gelernter J, Kaufman J (2013). Child abuse and epigenetic mechanisms of disease risk. Am J Prev Med.

[13] Marzi SJ, Sugden K, Arseneault L, Belsky DW, Burrage J, Corcoran DL, Danese A, Fisher HL, Hannon E, Moffitt TE, Odgers CL, Pariante C, Poulton R, Williams BS, Wong CCY, Mill J, Caspi A (2018). Analysis of DNA methylation in young people: limited evidence for an association between victimization stress and epigenetic variation in blood. Am J Psychiatry.

[14] Smith AK, Conneely KN, Kilaru V, Mercer KB, Weiss TE, Bradley B, Tang Y, Gillespie CF, Cubells JF, Ressler KJ (2011). Differential immune system DNA methylation and cytokine regulation in post-traumatic stress disorder. Am J Med Genet B Neuropsychiatr Genet.

[15] Baldwin JR, Reuben A, Newbury JB, Danese A (2019). Agreement between prospective and retrospective measures of childhood maltreatment: a systematic review and meta-analysis. JAMA Psychiat.

[16] Anda RF, Croft JB, Felitti VJ, Nordenberg D, Giles WH, Williamson DF, Giovino GA (1999). Adverse childhood experiences and smoking during adolescence and adulthood. J Am Med Assoc.

[17] Zeilinger S, Kühnel B, Klopp N, Baurecht H, Kleinschmidt A, Gieger C, Weidinger S, Lattka E, Adamski J, Peters A, Strauch K, Waldenberger M, Illig T (2013). Tobacco smoking leads to extensive genome-wide changes in DNA methylation. PLoS ONE.

[18] Ciccarone F, Tagliatesta S, Caiafa P, Zampieri M (2018). DNA methylation dynamics in aging: how far are we from understanding the mechanisms?. Mech Ageing Dev.

[19] Lawson GM, Camins JS, Wisse L, Wu J, Duda JT, Cook PA, Gee JC, Farah MJ (2017). Childhood socioeconomic status and childhood maltreatment: distinct associations with brain structure. PLoS ONE.

[20] McLaughlin KA, Sheridan MA, Humprehys KL, Belsky J, Ellis BJ (2021). The value of dimensional models of early experience: thinking clearly about concepts and categories. Perspect Psychol Sci.

[21] McLaughlin KA, Sheridan MA, Lambert HK (2014). Childhood adversity and neural development: deprivation and threat as distinct dimensions of early experience. Neurosci Biobehav Rev.

[22] Dunn EC, Soare TW, Zhu Y, Simpkin AJ, Suderman MJ, Klengel T, Smith ADAC, Ressler KJ, Relton CL (2019). Sensitive periods for the effect of childhood adversity on DNA methylation: results from a prospective, longitudinal study. Biol Psychiatry.

[23] Martins J, Czamara D, Sauer S, Rex-Haffner M, Dittrich K, Dörr P, de Punder K, Overfeld J, Knop A, Dammering F, Entringer S, Winter SM, Buss C, Heim C, Binder EB (2021). Childhood adversity correlates with stable changes in DNA methylation trajectories in children and converges with epigenetic signatures of prenatal stress. Neurobiol Stress.

[24] Weissman DG, Bitran D, Miller AB, Schaefer JD, Sheridan MA, McLaughlin KA (2019). Difficulties with emotion regulation as a transdiagnostic mechanism linking child maltreatment with the emergence of psychopathology. Dev Psychopathol.

[25] Jenness JL, Peverill M, Heleniak C, Robertson MM, Sambrook KA, Sheridan MA, McLaughlin KA (2021). Alterations in neural circuits underlying emotion regulation following child maltreatment: a mechanism underlying trauma-related psychopathology. Psychol Med.

[26] Bifulco A, Brown GW, Harris TO (1994). Childhood Experience of Care and Abuse (CECA): a retrospective interview measure. J Child Psychol Psychiatry.

[27] Raviv A, Raviv A, Shimoni H, Fox NA, Leavitt LA (1999). Children’s self-report of exposure to violence and its relation to emotional distress. J Appl Dev Psychol.

[28] Bernstein DP, Ahluvalia T, Pogge D, Handelsman L (1997). Validity of the Childhood Trauma Questionnaire in an adolescent psychiatric population. J Am Acad Child Adolesc Psychiatry.

[29] Steinberg AM, Brymer MJ, Kim S, Briggs EC, Ippen CG, Ostrowski SA, Gully KJ, Pynoos RS (2013). Psychometric properties of the UCLA PTSD reaction index: part I. J Trauma Stress.

[30] Straus MA, Hamby SL, Finkelhor D, Moore DW, Runyan D (1998). Identification of child maltreatment with the Parent-Child Conflict Tactics Scales: development and psychometric data for a national sample of American parents. Child Abuse Negl.

[31] Finkelhor D, Hamby SL, Ormrod R, Turner H (2005). The Juvenile Victimization Questionnaire: reliability, validity, and national norms. Child Abuse Negl.

[32] Blumberg SJ, Bialostosky K, Hamilton WL, Briefel RR (1999). The effectiveness of a short form of the Household Food Security Scale. Am J Public Health.

[33] Mott FL (2004). The utility of the HOME-SF scale for child development research in a large national longitudinal survey: the National Longitudinal Survey of Youth 1979 cohort. Parenting.

[34] Aryee MJ, Jaffe AE, Corrada-Bravo H, Ladd-Acosta C, Feinberg AP, Hansen KD, Irizarry RA (2014). Minfi: a flexible and comprehensive Bioconductor package for the analysis of Infinium DNA methylation microarrays. Bioinformatics.

[35] Johnson SB, Riis JL, Noble KG (2016). State of the art review: poverty and the developing brain. Pediatrics.

[36] Titus AJ, Gallimore RM, Salas LA, Christensen BC (2017). Cell-type deconvolution from DNA methylation: a review of recent applications. Hum Mol Genet.

[37] Houseman EA, Kile ML, Christiani DC, Ince TA, Kelsey KT, Marsit CJ (2016). Reference-free deconvolution of DNA methylation data and mediation by cell composition effects. BMC Bioinform.

[38] Barfield RT, Almli LM, Kilaru V, Smith AK, Mercer KB, Duncan R, Klengel T, Mehta D, Binder EB, Epstein MP, Ressler KJ, Conneely KN (2014). Accounting for population stratification in DNA methylation studies. Genet Epidemiol.

[39] Achenbach TM (1991). Integrative guide for the 1991 CBCL/4-18, YSR and TRF profiles.

[40] Barfield RT, Kilaru V, Smith AK, Conneely KN (2012). CpGassoc: an R function for analysis of DNA methylation microarray data. Bioinformatics.

[41] Johnson WE, Li C, Rabinovic A (2007). Adjusting batch effects in microarray expression data using empirical Bayes methods. Biostatistics.

[42] Liu PZ, Nusslock R (2018). How stress gets under the skin: early life adversity and glucocorticoid receptor epigenetic regulation. Curr Genom.

[43] Parade SH, Parent J, Rabemananjara K, Seifer R, Marsit CJ, Yang B-Z, Zhang H, Tyrka AR (2017). Change in FK506 binding protein 5 (FKBP5) methylation over time among preschoolers with adversity. Dev Psychopathol.

[44] Ramo-Fernández L, Boeck C, Koenig AM, Schury K, Binder EB, Gündel H, Fegert JM, Karabatsiakis A, Kolassa I-T (2019). The effects of childhood maltreatment on epigenetic regulation of stress-response associated genes: an intergenerational approach. Sci Rep.

[45] Murgatroyd C, Patchev AV, Wu Y, Micale V, Bockmühl Y, Fischer D, Holsboer F, Wotjak CT, Almeida OF, Spengler D (2009). Dynamic DNA methylation programs persistent adverse effects of early-life stress. Nat Neurosci.

[46] Provenzi L, Giorda R, Beri S, Montirosso R (2016). SLC6A4 methylation as an epigenetic marker of life adversity exposures in humans: a systematic review of literature. Neurosci Biobehav Rev.

[47] Schechter DS, Moser DA, Pointet VC, Aue T, Stenz L, Paoloni-Giacobino A, Adouan W, Manini A, Suardi F, Vital M, Rossignol AS, Cordero MI, Rothenberg M, Ansermet F, Serpa SR, Dayer AG (2017). The association of serotonin receptor 3A methylation with maternal violence exposure, neural activity, and child aggression. Behav Brain Res.

[48] Checknita D, Ekström TJ, Comasco E, Nilsson KW, Tiihonen J, Hodgins S (2018). Associations of monoamine oxidase A gene first exon methylation with sexual abuse and current depression in women. J Neural Transm.

[49] Kundakovic M, Gudsnuk K, Herbstman JB, Tang D, Perera FP, Champagne FA (2015). DNA methylation of BDNF as a biomarker of early-life adversity. Proc Natl Acad Sci U S A.

[50] Smearman EL, Almli LM, Conneely KN, Brody GH, Sales JM, Bradley B, Ressler KJ, Smith AK (2016). Oxytocin receptor genetic and epigenetic variations: association with child abuse and adult psychiatric symptoms. Child Dev.

[51] Phipson B, Maksimovic J, Oshlack A (2016). missMethyl: an R package for analyzing data from Illumina’s HumanMethylation450 platform. Bioinformatics.

[52] Stylli SS, Stacey T, Verhagen AM, Pass I, Courtneidge SA, Lock P (2009). Nck adaptor proteins link Tks5 to invadopodia actin regulation and ECM degradation. J Cell Sci.

[53] Blouw B, Seals DF, Pass I, Diaz B, Courtneidge SA (2008). A role for the podosome/invadopodia scaffold protein Tks5 in tumor growth in vivo. Eur J Cell Biol.

[54] Li CM-C, Chen G, Dayton TL, Kim-Kiselak C, Hoersch S, Whittaker CA, Bronson RT, Beer DG, Winslow MM, Jacks T (2013). Differential Tks5 isoform expression contributes to metastatic invasion of lung adenocarcinoma. Genes Dev.

[55] Malnic B, Godfrey PA, Buck LB (2004). The human olfactory receptor gene family. Proc Natl Acad Sci U S A.

[56] Chen Y, Li X, Kobayashi I, Tsao D, Mellman TA (2016). Expression and methylation in posttraumatic stress disorder and resilience; evidence of a role for odorant receptors. Psychiatry Res.

[57] Yan W, Wu F, Morser J, Wu Q (2000). Corin, a transmembrane cardiac serine protease, acts as a pro-atrial natriuretic peptide-converting enzyme. Proc Natl Acad Sci U S A.

[58] Ikeda Y, Imai Y, Kumagai H, Nosaka T, Morikawa Y, Hisaoka T, Manabe I, Maemura K, Nakaoka T, Imamura T, Miyazono K, Komuro I, Nagai R, Kitamura T (2004). Vasorin, a transforming growth factor β-binding protein expressed in vascular smooth muscle cells, modulates the arterial response to injury in vivo. Proc Natl Acad Sci U S A.

[59] Bonnet A-L, Chaussain C, Broutin I, Rochefort GY, Schrewe H, Gaucher C (2018). From vascular smooth muscle cells to folliculogenesis: what about vasorin?. Front Med.

[60] Eriksson A, Williams MJ, Voisin S, Hansson I, Krishnan A, Philippot G, Yamskova O, Herisson FM, Dnyansagar R, Moschonis G, Manios Y, Chrousos GP, Olszewski PK, Frediksson R, Schiöth HB (2015). Implication of coronin 7 in body weight regulation in humans, mice and flies. BMC Neurosci.

[61] Koyanagi M, Nakabayashi K, Fujimoto T, Gu N, Baba I, Takashima Y, Doi K, Harada H, Kato N, Sasazuki T, Shirasawa S (2008). ZFAT expression in B and T lymphocytes and identification of ZFAT-regulated genes. Genomics.

[62] Dube SR, Fairweather D, Pearson WS, Felitti VJ, Anda RF, Croft JB (2009). Cumulative childhood stress and autoimmune diseases in adults. Psychosom Med.

[63] Lacey RE, Pereira SMP, Li L, Danese A (2020). Adverse childhood experiences and adult inflammation: single adversity, cumulative risk and latent class approaches. Brain Behav Immun.

[64] Kuan P-F, Waszczuk MA, Kotov R, Marsit CJ, Guffanti G, Gonzalez A, Yang X, Koenen K, Bromet E, Luft BJ (2017). An epigenome-wide DNA methylation study of PTSD and depression in World Trade Center responders. Transl Psychiatry.

[65] Rutten BP, Vermetten E, Vinkers CH, Ursini G, Daskalakis NP, Pishva E, de Nijs L, Houtepen LC, Eijssen L, Jaffe AE, Kenis G, Viechtbauer W, van den Hove D, Schraut KG, Lesch K-P, Kleiman JE, Hyde TM, Weinberger DR, Schalkwyk L, Lunnon K, Mill J, Cohen H, Yehuda R, Baker DG, Maihofer AX, Nievergelt CM, Geuze E, Boks MPM (2018). Longitudinal analyses of the DNA methylome in deployed military servicemen identify susceptibility loci for post-traumatic stress disorder. Mol Psychiatry.

[66] Mulder RH, Neumann A, Cecil CAM, Walton E, Houtepen LC, Simpkin AJ, Rijlaarsdam J, Heijmans BT, Gaunt TR, Felix JF, Jaddoe VWV, Bakermans-Kranenburg MJ, Tiemeier H, Relton CL, van Ijzendoorn MH, Suderman M (2021). Epigenome-wide change and variation in DNA methylation in childhood: trajectories from birth to late adolescence. Hum Mol Genet.

[67] Min JL, Hemani G, Hannon E, Dekkers KF, Castillo-Fernandez J, Luijk R, Carnero-Montoro E, Lawson DJ, Burrows K, Suderman M, Bretherick AD, Richardson TG, Klughammer J, Iotchkova V, Sharp G, Al Khleifat A, Shatunov A, Iacoangeli A, McArdle WL, Ho KM, Kumar A, Söderhäll C, Soriano-Tárraga C, Giralt-Steinhauer E, Kazmi N, Mason D, McRae AF, Corcoran DL, Sugden K, Kasela S, Cardona A, Day FR, Cugliari G, Viberti C, Guarrera S, Lerro M, Gupta R, Bollepalli S, Mandaviya P, Zeng Y, Clarke TK, Walker RM, Schmoll V, Czamara D, Ruiz-Arenas C, Rezwan FI, Marioni RE, Lin T, Awaloff Y, Germain M, Aïssi D, Zwamborn R, van Eijk K, Dekker A, van Dongen J, Hottenga JJ, Willemsen G, Xu CJ, Barturen G, Català-Moll F, Kerick M, Wang C, Melton P, Elliott HR, Shin J, Bernard M, Yet I, Smart M, Gorrie-Stone T; BIOS Consortium, Shaw C, Al Chalabi A, Ring SM, Pershagen G, Melén E, Jiménez-Conde J, Roquer J, Lawlor DA, Wright J, Martin NG, Montgomery GW, Moffitt TE, Poulton R, Esko T, Milani L, Metspalu A, Perry JRB, Ong KK, Wareham NJ, Matullo G, Sacerdote C, Panico S, Caspi A, Arseneault L, Gagnon F, Ollikainen M, Kaprio J, Felix JF, Rivadeneira F, Tiemeier H, van IJzendoorn MH, Uitterlinden AG, Jaddoe VWV, Haley C, McIntosh AM, Evans KL, Murray A, Räikkönen K, Lahti J, Nohr EA, Sørensen TIA, Hansen T, Morgen CS, Binder EB, Lucae S, Gonzalez JR, Bustamante M, Sunyer J, Holloway JW, Karmaus W, Zhang H, Deary IJ, Wray NR, Starr JM, Beekman M, van Heemst D, Slagboom PE, Morange PE, Trégouët DA, Veldink JH, Davies GE, de Geus EJC, Boomsma DI, Vonk JM, Brunekreef B, Koppelman GH, Alarcón-Riquelme ME, Huang RC, Pennell CE, van Meurs J, Ikram MA, Hughes AD, Tillin T, Chaturvedi N, Pausova Z, Paus T, Spector TD, Kumari M, Schalkwyk LC, Visscher PM, Davey Smith G, Bock C, Gaunt TR, Bell JT, Heijmans BT, Mill J, Relton CL. Genomic and phenotypic insights from an atlas of genetic effects on DNA methylation. Nat Genet. 2021;53:1311–1321. 10.1038/s41588-021-00923-x PMC7612069 34493871

[68] Warrier V, Kwong ASF, Luo M, Dalvie S, Croft J, Sallis HM, Baldwin J, Munafò MR, Nievergelt CM, Grant AJ, Burgess S, Moore TM, Barzilay R, McIntosh A, van IJzendoorn MH, Cecil CAM (2021). Gene-environment correlations and causal effects of childhood maltreatment on physical and mental health: a genetically informed approach. Lancet Psychiatry.

[69] Czamara D, Tissink E, Tuhkanen J, Martins J, Awaloff Y, Drake AJ, Khulan B, Palotie A, Winter SM, Nemeroff CB, Craighead WE, Dunlop BW, Mayberg HS, Kinkead B, Mathew SJ, Iosifescu DV, Neylan TC, Heim CM, Lahti J, Eriksson JG, Räikkönen K, Ressler KJ, Provençal N, Binder EB (2021). Combined effects of genotype and childhood adversity shape variability of DNA methylation across age. Transl Psychiatry.

[70] The GoDMC Database. http://mqtldb.godmc.org.uk. Accessed 27 Dec 2021.

