# Supplementary material for: Epigenetics of early-life adversity in youth: cross-sectional and longitudinal associations
Source: Clin Epigenetics. 2022 Apr 8;14:48. doi: 10.1186/s13148-022-01269-9 (PMC8994405; doi:10.1186/s13148-022-01269-9)

**Additional File 1**

**Supplemental Methods**

**Participants and Procedure**

Youths aged 8-16 years and a caregiver (parent or guardian) were recruited to participate in a longitudinal study examining early life adversity (ELA), emotion regulation, and psychopathology. Between January 2015 and January 2017, 262 youths were enrolled from the community in Seattle, WA [1]. Youths and caregivers were recruited for participation at schools, after-school and prevention programs, adoption programs, food banks, shelters, parenting programs, medical clinics, and the general community. In order to recruit a sample with variation in ELA experiences, the research team recruited from neighborhoods with high violent crime, clinics serving a predominantly low-socioeconomic status catchment area, and agencies working with families who have been victims of violence (e.g., domestic violence shelters, programs for parents mandated to receive intervention by Child Protective Services). Participants were invited to complete a baseline assessment and to return to the research laboratory approximately 2 years later for a follow-up assessment. Study procedures were approved by the University of Washington Institutional Review Board. Caregivers provided written informed consent; youths provided written assent.

**ELA**

At baseline, youths completed two interviews assessing lifetime experiences of ELA with a trained member of the research team. The Childhood Experiences of Care and Abuse (CECA) interview assesses caregiving experiences, including physical, sexual, and emotional abuse and emotional and physical neglect [2]. Inter-rater reliability for maltreatment reports is excellent, and validation studies suggest high agreement between siblings on maltreatment reports [3]. Presence of physical or sexual abuse were each based on youth report of these experiences, and validated thresholds were used to identify the presence of emotional abuse (maternal or paternal caregiver antipathy score $\geq$25) and neglect (maternal caregiver neglect score $\geq$24 or paternal caregiver neglect score $\geq$22) [4]. The Violence Exposure Scale for Children-Revised (VEX-R) [5,6] assesses the frequency of exposure to different forms of violence. Children are presented with a cartoon and caption depicting a child of the same sex witnessing a type of violence (e.g., “Chris sees a person slap another person really hard”) and experiencing that same type of violence (e.g., “A person slaps Chris really hard”). Children are then asked to report how frequently they have witnessed or experienced that type of violence (e.g., “How many times have you seen a person slap another person really hard?”; “How many times has a person slapped you really hard?”) on a Likert scale ranging from 0 (Never) to 3 (Lots of times). The VEX-R demonstrates good reliability and validity in child samples [5,6]. We created a violence exposure composite on the VEX-R by summing the number of distinct forms of violence the child experienced or witnessed firsthand. Only items clearly reflecting violence were included (i.e., either experiencing or witnessing being pushed or shoved really hard, slapped really hard, beaten up, having a gun or knife pointed at them, and corporal punishment); items that did not clearly reflect violence were not included in the count (e.g., seeing someone selling drugs).

Youths additionally completed two self-report measures assessing lifetime experiences of child maltreatment and trauma. The Childhood Trauma Questionnaire (CTQ) is a 28-item scale that assesses the frequency of maltreatment during childhood, including physical, sexual, and emotional abuse and physical and emotional neglect. The CTQ has excellent psychometric properties including internal consistency, test-retest reliability, and convergent and discriminant validity with interviews and clinician reports of maltreatment [7,8]. Validated thresholds for physical abuse (subscale score $\geq$8), sexual abuse (subscale score $\geq$8), and emotional abuse (subscale score $\geq$10) were applied here in evaluating abuse exposure based on the CTQ [9]. These widely used subscale thresholds were identified in prior research with the CTQ as providing the best balance of sensitivity and specificity [9]. The UCLA Posttraumatic Stress Disorder Reaction Index (PTSD-RI) includes a trauma screen that assesses exposure to numerous traumatic events, including physical abuse, sexual abuse, and domestic violence. The PTSD-RI has good internal consistency and convergent validity [10].

At baseline, caregivers completed three self-report measures of youths’ experiences of child maltreatment and trauma: the Conflict Tactics Scale-Parent Child Version (CTS) [11], the Juvenile Victimization Questionnaire (JVQ) lifetime caregiver report [12], and the caregiver version of the PTSD-RI [10]. The CTS includes 22 items assessing caregiver responses to child disobedience or misbehavior in the past year. Caregivers indicate how frequently they have used each strategy (e.g., shook him/her) on a Likert scale ranging from 0 (“This has never happened”) to 6 (“More than 20 times in the past year”) and can also indicate if they have used the strategy in the past but not in the last year. The CTS has adequate reliability and good discriminant and construct validity [11]. The JVQ includes 34 items assessing experiences of crime, child maltreatment, peer and sibling victimization, sexual victimization, and witnessing and indirect victimization, and it has excellent psychometric properties, including test-retest reliability and construct validity [12]. Caregivers endorsed whether their child had experienced each event in his/her lifetime. Caregivers also completed the trauma screen included in the PTSD-RI, described above, for their child. A trained interviewer followed up with caregivers if they endorsed any form of abuse or domestic violence to gather additional information about the experience.

Food insecurity in the past 12 months was assessed using a set of 4 items drawn from the short form of the U.S. Department of Agriculture’s Food Security Scale [13]. These four items constitute a validated measure of food insecurity that has been used in epidemiological surveys of youth psychopathology (e.g., the National Comorbidity Survey Replication – Adolescent Supplement [14]). Caregivers completed two dichotomous items indicating whether they had ever been hungry but did not eat because they could not afford enough food and whether they had ever eaten less than they thought they should because there was not enough money to buy food. Two additional items assessed how often caregivers did not have enough money to buy food and could not afford to buy balanced meals in the past 12 months.

To assess the degree of cognitive stimulation in the home environment, caregivers completed the Home Observation for Measurement of the Environment-Short Form (HOME-SF) [15]. The HOME-SF has slightly different versions for youths aged 6-9 and 10-15 years; 16 items are identical across these age ranges. As our sample included both of these age windows, we only used the 16 questions present in the HOME-SF for both younger and older children. This assessment included items that assess cognitive stimulation and exposure to varied learning experiences. Example items included: “About how many books does your child have?”; “How many times does your child get out of the house per week for activities (e.g., sports, extracurricular activities, activities with the family)?”; and “Did you and/or your partner teach your child numbers at home?”. The measure was scored using the cut-offs used in the original HOME assessment, where one point is assigned for each item where age-appropriate experiences are met (e.g., 1 point is assigned if a child leaves the house at least once a week for an activity; 0 is assigned if the child leaves the house less than once a week for an activity), for a total possible score of 16.

At the follow-up assessment approximately 2 years after baseline, youths and caregivers completed the above interviews and/or questionnaires with respect to experiences that occurred since the baseline assessment. At both baseline and follow-up, Child Protective Services were alerted to any cases of child abuse or neglect that had not previously been reported to the proper authorities.

Lifetime experience of abuse at baseline included threat-related experiences of physical abuse, sexual abuse, and/or emotional abuse, and was determined by combining youth and caregiver baseline reports using an “or” rule (i.e., a particular ELA was coded present if either the caregiver or the child endorsed it). Physical and sexual abuse were coded present if the youth endorsed these experiences in the CECA interview, scored above the validated thresholds on the CTQ sub-scales for physical and sexual abuse [9], or if the parent endorsed these experiences on the CTS, JVQ, or PTSD-RI. Emotional abuse was coded based on youth report only and was considered present if the youth scored above the validated thresholds on either the CECA interview [4] or CTQ subscale for emotional abuse [9]. Experience of abuse (i.e., physical, sexual, or emotional abuse) that occurred over follow-up was determined in a similar way using youth and caregiver reports at the follow-up assessment.

Lifetime experience of neglect at baseline included deprivation-related experiences of emotional and/or physical neglect, and was based on youth report. Neglect was coded based on the CECA interview using validated thresholds [4]. We elected to use the CECA rather than the CTQ for assessing emotional and physical neglect, as this measure more closely aligns with accepted definitions of neglect [16] by assessing neglectful behaviors (e.g., “She would leave me unsupervised before the age of 10”) as compared to the CTQ, which focuses largely on appraisals (e.g., “My family was a source of strength and support”). As with abuse, experience of neglect (i.e., emotional or physical neglect) that occurred over follow-up was determined in a similar way using youth reports at the follow-up assessment.

In addition to dichotomous measures of abuse and neglect, we calculated continuous threat and deprivation composites reflecting the frequency and severity of those experiences. The threat experiences composite summed the total number of threat experiences endorsed by the child and/or caregiver. Threat experiences included physical abuse, sexual abuse, and emotional abuse (i.e., the experiences comprising the abuse variable), in addition to domestic violence and exposure to other forms of interpersonal violence. Domestic violence was considered present if it was endorsed by the youth on the CECA interview or the PTSD-RI or by the caregiver on the JVQ or PTSD-RI. Finally, the frequencies of exposure to different forms of interpersonal violence reported by the child on the VEX-R were summed.

We created a deprivation composite using the same procedures. The deprivation experiences composite included the experiences comprising the neglect variable, in addition to physical neglect from the CTQ, food insecurity, and an absence of cognitive stimulation (i.e., cognitive deprivation). Physical neglect from the CTQ was coded present if children scored above a validated threshold (subscale score $\geq$8) [9]. Food insecurity was coded as present if caregivers endorsed being hungry but not eating because they could not afford food or eating less than they thought they should because there was not enough money to buy food. Cognitive stimulation was assessed using the HOME-SF, which does not have an established cut-point for stimulation low enough to be considered deprivation. As such, we coded youths in the bottom quartile of this measure in our sample as experiencing cognitive deprivation.

**References**

1. Weissman DG, Bitran D, Miller AB, Schaefer JD, Sheridan MA, McLaughlin KA. Difficulties with emotion regulation as a transdiagnostic mechanism linking child maltreatment with the emergence of psychopathology. Dev Psychopathol. 2019;31:899-915.

2. Bifulco A, Brown GW, Harris TO. Childhood Experience of Care and Abuse (CECA): a retrospective interview measure. J Child Psychol Psychiatry 1994;35:1419-1435.

3. Bifulco A, Brown G, Lillie A, Jarvis J. (1997). Memories of childhood neglect and abuse: corroboration in a series of sisters. J Child Psychol Psychiatry 1997;38:365-374.

4. Bifulco A, Bernazzani O, Moran P, Jacobs C. The childhood experience of care and abuse questionnaire (CECA.Q): validation in a community series. Br J Clin Psychol. 2005;44:563-581.

5. Raviv A, Raviv A, Shimoni H, Fox NA, Leavitt LA. Children's self-report of exposure to violence and its relation to emotional distress. J Appl Dev Psychol. 1999;20:337-353.

6. Raviv A, Erel O, Fox NA, Leavitt LA, Raviv A, Dar I, Shahinfar A, Greenbaum CW. Individual measurement of exposure to everyday violence among elementary school children across various settings. J Community Psychol. 2001;29:117-140.

7. Bernstein DP, Ahluvalia T, Pogge D, Handelsman L. Validity of the Childhood Trauma Questionnaire in an adolescent psychiatric population. J Am Acad Child Adolesc Psychiatry 1997;36:340-348.

8. Bernstein DP, Fink L, Handelsman L, Foote J, Lovejoy M, Wenzel K, Sapareto E, Ruggiero J. Initial reliability and validity of a new retrospective measure of child abuse and neglect. Am J Psychiatry 1994;151:1132-1136.

9. Walker EA, Unutzer J, Rutter C, Gelfand A, Saunders K, VonKorff M, Koss MP, Katon W. Costs of health care use by women HMO members with a history of childhood abuse and neglect. Arch Gen Psychiatry 1999;56:609-613.

10. Steinberg AM, Brymer MJ, Kim S, Briggs EC, Ippen CG, Ostrowski SA, Gully KJ, Pynoos RS. Psychometric properties of the UCLA PTSD Reaction Index: part I. J Trauma Stress 2013;26:1-9.

11. Straus MA, Hamby SL, Finkelhor D, Moore DW, Runyan D. Identification of child maltreatment with the Parent-Child Conflict Tactics Scales: development and psychometric data for a national sample of American parents. Child Abuse Negl. 1998;22:249-270.

12. Finkelhor D, Hamby SL, Ormrod R, Turner H. The Juvenile Victimization Questionnaire: reliability, validity, and national norms. Child Abuse Negl. 2005;29:383-412.

13. Blumberg SJ, Bialostosky K, Hamilton WL, Briefel RR. The effectiveness of a short form of the Household Food Security Scale. Am J Public Health 1999;89:1231-1234.

14. McLaughlin KA, Green JG, Alegría M, Costello EJ, Gruber MJ, Sampson NA, Kessler RC. Food insecurity and mental disorders in a national sample of US adolescents. J Am Acad Child Adolesc Psychiatry 2012;51:1293-1303.

15. Mott FL. The utility of the HOME-SF scale for child development research in a large national longitudinal survey: the National Longitudinal Survey of Youth 1979 cohort. Parenting 2004;4:259-270.

16. Straus MA, Kantor GK. Definition and measurement of neglectful behavior: some principles and guidelines. Child Abuse Negl. 2005;29:19-29.

**Table S1.** Characteristics of participants who were included in the analytic sample compared to all cohort participants who were eligible to provide saliva samples for epigenetic analyses.

| **Characteristic** | **Participants in the Analytic Sample**  **(*n*=113)** | **Eligible Participants in the Cohort**  **(*n=*161)** |
| --- | --- | --- |
|  | *M* (*SD*) or % (*n*) | *M* (*SD*) or % (*n*) |
| Age at baseline, years | 12.2 (2.5) | 12.5 (2.7) |
| Female sex | 42.5 (48) | 47.8 (77) |
| Race/ethnicity |  |  |
| White | 44.2 (50) | 42.9 (69) |
| Black | 21.2 (24) | 24.2 (39) |
| Latino | 11.5 (13) | 9.9 (16) |
| Asian | 10.6 (12) | 11.2 (18) |
| Other | 12.4 (14) | 11.8 (19) |
| Family income-to-needs ratio, baseline | 3.9 (2.8) | 3.7 (2.8) |
| Family income-to-needs ratio, follow-up | 3.7 (2.8) | 3.8 (2.8) |
|  |  |  |
| **Early life adversity** |  |  |
| Lifetime experience of abuse, baseline | 52.2 (59) | 54.7 (88) |
| Lifetime experience of neglect, baseline | 26.5 (30) | 27.3 (44) |
| Lifetime threat composite, baseline | 4.9 (3.7) | 5.0 (3.8) |
| Lifetime deprivation composite, baseline | 0.9 (1.1) | 0.9 (1.1) |
| Experience of abuse during follow-up | 31.0 (35) | 22.4 (36) |
| Experience of neglect during follow-up | 11.5 (13) | 8.1 (13) |
| Threat composite during follow-up | 2.6 (2.7) | 2.1 (2.6) |
| Deprivation composite during follow-up | 0.9 (1.1) | 0.7 (1.0) |

*Note.* df=degrees of freedom, M=mean, SD=standard deviation.

**Table S2.** Effect estimates for the other ELA type for probes significantly associated with lifetime experience of abuse or neglect at baseline in cross-sectional epigenome-wide analyses.

| **Probe ID** | **Position** | **Gene** | **Gene Region Feature^†^** | **Coefficient (SE)** | **FDR** |
| --- | --- | --- | --- | --- | --- |
| **Effect estimates for neglect for probes significantly associated with lifetime experience of abuse** | | | | | |
| **Model 1^§^** | | | | | |
| cg08671764 | chr11:57996369 | *OR10Q1* | 1^st^ exon | 0.005 (0.003) | .799 |
| cg19454603 | chr11:107730446 | *SLC35F2* | TSS1500 | -0.003 (0.001) | .383 |
| cg05462437 | chr14:70528945 | *SLC8A3* | 5’UTR | 0.011 (0.004) | .554 |
| cg25625296 | chr1:1145848 | Unassigned | Unassigned | -0.015 (0.005) | .563 |
| cg18348616 | chr10:97470256 | *ENTPD1* | TSS1500 | -0.041 (0.019) | .687 |
| cg26357241 | chr1:109289876 | *STXBP3* | Body | -0.008 (0.002) | .440 |
| cg06383709 | chr16:26148977 | *HS3ST4* | 3’UTR | 0.007 (0.003) | .685 |
| cg00992846 | chr12:122906318 | *CLIP1* | 5’UTR | -0.001 (0.001) | .672 |
| cg16793662 | chr2:55407554 | *CLHC1* | Body | -0.027 (0.013) | .692 |
| cg07451097 | chr4:143641250 | *INPP4B* | 5’UTR | 0.024 (0.009) | .590 |
| cg10091662 | chr10:22609897 | *BMI1* | TSS200 | -0.006 (0.003) | .778 |
| cg15350036 | chr7:86973677 | *TP53TG1* | TSS1500 | -0.040 (0.010) | .362 |
| cg03727700 | chr10:115614022 | *DCLRE1A* | TSS1500 | -0.003 (0.001) | .362 |
| cg02047211 | chr17:78932697 | *RPTOR* | Body | 0.005 (0.002) | .672 |
| cg04866720 | chr2:150588473 | *LOC101929231* | Body | 0.005 (0.002) | .519 |
| **Model 2^¶^** |  |  |  |  |  |
| cg20241299 | chr10:105362799 | *SH3PXD2A* | Body | -0.012 (0.003) | .496 |
| cg08671764 | chr11:57996369 | *OR10Q1* | 1^st^ exon | -0.004 (0.003) | .841 |
| cg27152686 | chr4:47645625 | *CORIN* | Body | 0.030 (0.011) | .628 |
| cg24241897 | chr16:4421892 | *VASN*/*CORO7* | 1^st^ exon | 0.003 (0.001) | .274 |
|  |  |  |  |  |  |
| **Effect estimates for abuse for probes significantly associated with lifetime experience of neglect** | | | | | |
| **Model 1^§^** |  |  |  |  |  |
| cg00284420 | chr16:87311948 | Unassigned | Unassigned | -0.007 (0.002) | .104 |

*Note*. Chr=chromosome; FDR=false discovery rate; SE=standard error.

**^†^**Gene region feature category describing the CpG position from the UCSC Genome Browser. TSS200=0-200 bases upstream of the transcriptional start site (TSS); TSS1500=200-1500 bases upstream of the TSS; 5'UTR=within the 5' untranslated region (UTR), between the TSS and the ATG start site; Body=between the ATG and stop codon; irrespective of the presence of introns, exons, TSS, or promoters; 3'UTR=between the stop codon and poly A signal.

**^§^**Model 1 adjusted for age and income-to-needs ratio at baseline, sex, first five cell-type principal components, first five ancestry principal components, and random batch effects of DNA methylation measurement.

**^¶^**Model 2 adjusted for Model 1 covariates and lifetime abuse.

**Table S3.** Effect estimates for lifetime threat and deprivation composites for probes significantly associated with lifetime experience of abuse or neglect at baseline in cross-sectional epigenome-wide analyses.

| **Probe ID** | **Position** | **Gene** | **Gene Region Feature^†^** | **Coefficient (SE)** | **FDR** |
| --- | --- | --- | --- | --- | --- |
| **Effect estimates for threat for probes significantly associated with lifetime experience of abuse** | | | | | |
| **Model 1^§^** | | | | | |
| cg08671764 | chr11:57996369 | *OR10Q1* | 1^st^ exon | 0.001 (0.000) | .489 |
| cg19454603 | chr11:107730446 | *SLC35F2* | TSS1500 | -0.000 (0.000) | .444 |
| cg05462437 | chr14:70528945 | *SLC8A3* | 5’UTR | 0.002 (0.000) | .364 |
| cg25625296 | chr1:1145848 | Unassigned | Unassigned | -0.003 (0.001) | .309 |
| cg18348616 | chr10:97470256 | *ENTPD1* | TSS1500 | -0.009 (0.002) | .364 |
| cg26357241 | chr1:109289876 | *STXBP3* | Body | -0.001 (0.000) | .437 |
| cg06383709 | chr16:26148977 | *HS3ST4* | 3’UTR | 0.002 (0.000) | .347 |
| cg00992846 | chr12:122906318 | *CLIP1* | 5’UTR | -0.000 (0.000) | .443 |
| cg16793662 | chr2:55407554 | *CLHC1* | Body | -0.006 (0.002) | .364 |
| cg07451097 | chr4:143641250 | *INPP4B* | 5’UTR | 0.004 (0.001) | .353 |
| cg10091662 | chr10:22609897 | *BMI1* | TSS200 | -0.001 (0.000) | .561 |
| cg15350036 | chr7:86973677 | *TP53TG1* | TSS1500 | -0.006 (0.001) | .200 |
| cg03727700 | chr10:115614022 | *DCLRE1A* | TSS1500 | -0.000 (0.000) | .231 |
| cg02047211 | chr17:78932697 | *RPTOR* | Body | 0.001 (0.000) | .501 |
| cg04866720 | chr2:150588473 | *LOC101929231* | Body | 0.001 (0.000) | .327 |
| **Model 2^¶^** |  |  |  |  |  |
| cg20241299 | chr10:105362799 | *SH3PXD2A* | Body | 0.000 (0.001) | .999 |
| cg08671764 | chr11:57996369 | *OR10Q1* | 1^st^ exon | 0.001 (0.001) | .999 |
| cg27152686 | chr4:47645625 | *CORIN* | Body | -0.003 (0.002) | .999 |
| cg24241897 | chr16:4421892 | *VASN*/*CORO7* | 1^st^ exon | -0.000 (0.000) | .999 |
|  |  |  |  |  |  |
| **Effect estimates for deprivation for probes significantly associated with lifetime experience of neglect** | | | | | |
| **Model 1^§^** |  |  |  |  |  |
| cg00284420 | chr16:87311948 | Unassigned | Unassigned | -0.003 (0.001) | .222 |

*Note*. Chr=chromosome; FDR=false discovery rate; SE=standard error.

**^†^**Gene region feature category describing the CpG position from the UCSC Genome Browser. TSS200=0-200 bases upstream of the transcriptional start site (TSS); TSS1500=200-1500 bases upstream of the TSS; 5'UTR=within the 5' untranslated region (UTR), between the TSS and the ATG start site; Body=between the ATG and stop codon; irrespective of the presence of introns, exons, TSS, or promoters; 3'UTR=between the stop codon and poly A signal.

**^§^**Model 1 adjusted for age and income-to-needs ratio at baseline, sex, first five cell-type principal components, first five ancestry principal components, and random batch effects of DNA methylation measurement.

**^¶^**Model 2 adjusted for Model 1 covariates and lifetime deprivation composite.

**Table S4.** Probes significantly associated with lifetime experience of abuse and neglect at baseline in cross-sectional epigenome-wide analyses when adjusting for youth tobacco use.

| **Probe ID** | **Position** | **Gene** | **Gene Region Feature^†^** | **Coefficient (SE)** | **FDR^‡^** |
| --- | --- | --- | --- | --- | --- |
| **Lifetime experience of abuse** | | | | | |
| **Model 1^§^** |  |  |  |  |  |
| cg16793662 | chr2:55407554 | *CLHC1* | Body | -0.071 (0.012) | .017 |
| cg18348616 | chr10:97470256 | *ENTPD1* | TSS1500 | -0.105 (0.018) | .017 |
| cg08671764 | chr11:57996369 | *OR10Q1* | 1^st^ exon | 0.016 (0.003) | .017 |
| cg25625296 | chr1:1145848 | Unassigned | Unassigned | -0.029 (0.005) | .017 |
| cg26357241 | chr1:109289876 | *STXBP3* | Body | -0.012 (0.002) | .032 |
| cg19454603 | chr11:107730446 | *SLC35F2* | TSS1500 | -0.005 (0.001) | .032 |
| cg05462437 | chr14:70528945 | *SLC8A3* | 5’UTR | 0.021 (0.004) | .032 |
| cg00992846 | chr12:122906318 | *CLIP1* | 5’UTR | -0.003 (0.000) | .032 |
| cg07451097 | chr4:143641250 | *INPP4B* | 5’UTR | 0.048 (0.009) | .032 |
| cg06173663 | chr7:101579929 | *CUX1* | Body | -0.043 (0.008) | .044 |
| cg10091662 | chr10:22609897 | *BMI1* | TSS200 | -0.017 (0.003) | .044 |
| cg19467876 | chr13:102442957 | *FGF14* | Body | 0.033 (0.006) | .044 |
| cg01039441 | chr20:18257638 | Unassigned | Unassigned | -0.004 (0.001) | .044 |
| cg07971698 | chr3:126857489 | Unassigned | Unassigned | -0.036 (0.007) | .044 |
| cg06383709 | chr16:26148977 | *HS3ST4* | 3’UTR | 0.015 (0.003) | .044 |
| cg25796129 | chr17:9121696 | *NTN1* | Body | -0.028 (0.005) | .044 |
| cg07044380 | chr6:86218431 | *SNX14* | Body | -0.054 (0.010) | .044 |
| cg03727700 | chr7:86973677 | *DCLRE1A* | TSS1500 | -0.004 (0.001) | .044 |
| cg20866090 | chr3:188537167 | *LPP* | Body | 0.021 (0.004) | .044 |
| cg09218773 | chr11:619014 | *MUPCDH* | Body | -0.067 (0.013) | .044 |
| cg25214552 | chr4:127891291 | Unassigned | Unassigned | 0.012 (0.002) | .044 |
| cg02047211 | chr17:78932697 | *RPTOR* | Body | 0.011 (0.002) | .044 |
| cg17804100 | chr1:42277934 | *HIVEP3* | 5’UTR | -0.019 (0.004) | .044 |
| cg22219371 | chr6:45621648 | Unassigned | Unassigned | 0.022 (0.004) | .044 |
| cg02397934 | chr6:27662101 | Unassigned | Unassigned | -0.004 (0.001) | .044 |
| cg27152686 | chr4:47645625 | *CORIN* | Body | -0.055 (0.010) | .044 |
| cg06644978 | chr18:73202039 | Unassigned | Unassigned | 0.010 (0.002) | .044 |
| cg11318895 | chr6:30301535 | *TRIM39* | Body | 0.031 (0.006) | .044 |
| cg16112795 | chr8:40382156 | Unassigned | Unassigned | 0.071 (0.014) | .044 |
| cg04866720 | chr2:150588473 | *LOC101929231* | Body | 0.009 (0.002) | .044 |
| cg03158194 | chr2:203773242 | *WDR12* | Body | 0.011 (0.002) | .044 |
| cg24939196 | chr6:169588809 | Unassigned | Unassigned | -0.053 (0.010) | .047 |
| cg22198853 | chr6:1594411 | Unassigned | Unassigned | 0.050 (0.010) | .047 |
| cg27318050 | chr6:26458189 | *BTN2A1* | 5’UTR | -0.030 (0.006) | .047 |
| cg04530116 | chr2:55459572 | *RPS27A* | 5’UTR | -0.019 (0.004) | .048 |
| **Model 2^¶^** |  |  |  |  |  |
| cg20241299 | chr10:105362799 | *SH3PXD2A* | Body | 0.022 (0.004) | .010 |
| cg27152686 | chr4:47645625 | *CORIN* | Body | -0.071 (0.011) | .010 |
| cg24241897 | chr16:4421892 | *VASN*/*CORO7* | 1^st^ exon | -0.003 (0.001) | .012 |
| cg08671764 | chr11:57996369 | *OR10Q1* | 1^st^ exon | 0.018 (0.003) | .012 |
| cg19735627 | chr14:70233737 | *SFRS5* | TSS200 | -0.003 (0.001) | .040 |
| cg16793662 | chr2:55407554 | *CLHC1* | Body | -0.072 (0.013) | .040 |
| cg12610585 | chr1:85041397 | *CTBS* | TSS1500 | 0.009 (0.002) | .040 |
| cg26379012 | chr17:77964070 | *TBC1D16* | Body | 0.004 (0.001) | .049 |
|  |  |  |  |  |  |
| **Lifetime experience of neglect** | | | | | |
| **Model 1^§^** |  |  |  |  |  |
| cg00284420 | chr16:87311948 | Unassigned | Unassigned | -0.009 (0.001) | .002 |

*Note*. Chr=chromosome; FDR=false discovery rate; SE=standard error.

**^†^**Gene region feature category describing the CpG position from the UCSC Genome Browser. TSS200=0-200 bases upstream of the transcriptional start site (TSS); TSS1500=200-1500 bases upstream of the TSS; 5'UTR=within the 5' untranslated region (UTR), between the TSS and the ATG start site; Body=between the ATG and stop codon; irrespective of the presence of introns, exons, TSS, or promoters; 3'UTR=between the stop codon and poly A signal.

**^‡^**Probes significant at FDR<.05.

**^§^**Model 1 adjusted for age and income-to-needs ratio at baseline, sex, the first five cell-type principal components, the first five ancestry principal components, and random batch effects of DNA methylation measurement.

**^¶^**Model 2 adjusted for Model 1 covariates and lifetime neglect.

**Table S5.** Gene ontology terms uniquely associated with experiences of abuse or neglect in cross-sectional and longitudinal analyses.

| **GO** | **ONT** | **Term** | **N** | **DE** | ***p-*DE** | **FDR^†^** |
| --- | --- | --- | --- | --- | --- | --- |
| **Experience of abuse** | | | | | | |
| GO:0005654 | CC | nucleoplasm | 3474 | 1218.6 | 7.4E-10 | **1.7E-05** |
| GO:0044428 | CC | nuclear part | 4843 | 1536.6 | 4.7E-08 | **5.4E-04** |
| GO:0031981 | CC | nuclear lumen | 4454 | 1414.3 | 8.4E-08 | **6.4E-04** |
| GO:0009057 | CC | nucleus | 7667 | 2343.4 | 2.2E-07 | **9.9E-04** |
| GO:0005634 | BP | macromolecule catabolic process | 1378 | 497.7 | 2.1E-07 | **9.9E-04** |
| GO:0044265 | BP | cellular macromolecule catabolic process | 1144 | 417.7 | 2.7E-07 | **1.0E-03** |
| GO:0031974 | CC | membrane-enclosed lumen | 5594 | 1742.9 | 5.5E-07 | **1.4E-03** |
| GO:0043233 | CC | organelle lumen | 5594 | 1742.9 | 5.5E-07 | **1.4E-03** |
| GO:0070013 | CC | intracellular organelle lumen | 5594 | 1742.9 | 5.5E-07 | **1.4E-03** |
| GO:0043231 | CC | intracellular membrane-bounded organelle | 11167 | 3380.8 | 1.3E-06 | **2.9E-03** |
| GO:0044446 | CC | intracellular organelle part | 9565 | 2927.9 | 9.7E-06 | **2.0E-02** |
| GO:0009894 | BP | regulation of catabolic process | 975 | 362.2 | 1.1E-05 | **2.0E-02** |
| GO:0044257 | BP | cellular protein catabolic process | 755 | 286.3 | 1.1E-05 | **2.0E-02** |
| GO:0031461 | CC | cullin-RING ubiquitin ligase complex | 158 | 75.8 | 1.4E-05 | **2.2E-02** |
| GO:1902494 | CC | catalytic complex | 1352 | 479.7 | 2.1E-05 | **3.1E-02** |
| GO:0030163 | BP | protein catabolic process | 913 | 337.8 | 2.8E-05 | **4.0E-02** |
|  |  |  |  |  |  |  |
| **Experience of neglect** | | | | | | |
| GO:0050806 | BP | positive regulation of synaptic transmission | 168 | 38.0 | 3.3E-04 | 1 |
| GO:0099537 | BP | trans-synaptic signaling | 707 | 115.5 | 5.7E-04 | 1 |
| GO:0007268 | BP | chemical synaptic transmission | 699 | 113.5 | 7.2E-04 | 1 |
| GO:0098916 | BP | anterograde trans-synaptic signaling | 699 | 113.5 | 7.2E-04 | 1 |
| GO:0099536 | BP | synaptic signaling | 713 | 115.5 | 8.0E-04 | 1 |
| GO:0099177 | BP | regulation of trans-synaptic signaling | 435 | 77.0 | 2.0E-03 | 1 |
| GO:0030673 | CC | axolemma | 14 | 7.0 | 2.5E-03 | 1 |
| GO:0050804 | BP | modulation of chemical synaptic transmission | 434 | 76.0 | 2.8E-03 | 1 |
| GO:0002017 | BP | regulation of blood volume by renal aldosterone | 2 | 2.0 | 5.2E-03 | 1 |
| GO:0098563 | CC | intrinsic component of synaptic vesicle membrane | 46 | 12.0 | 5.6E-03 | 1 |

*Note*. GO=Gene ontology term; ONT=Ontology that the GO term belongs to: BP (biological process), CC (Cellular Component), MF (Molecular function); N=number of genes in the GO term; DE=number of genes differently methylated; *p*-DE=*p*-value for over-representation of the GO term; FDR=false discovery rate.

**^†^**Bold terms significant at FDR<.05.

**Fig. S1.** Box plots of sex-stratified DNA methylation β-values that were significantly associated with lifetime abuse or neglect in cross-sectional epigenome-wide analyses.


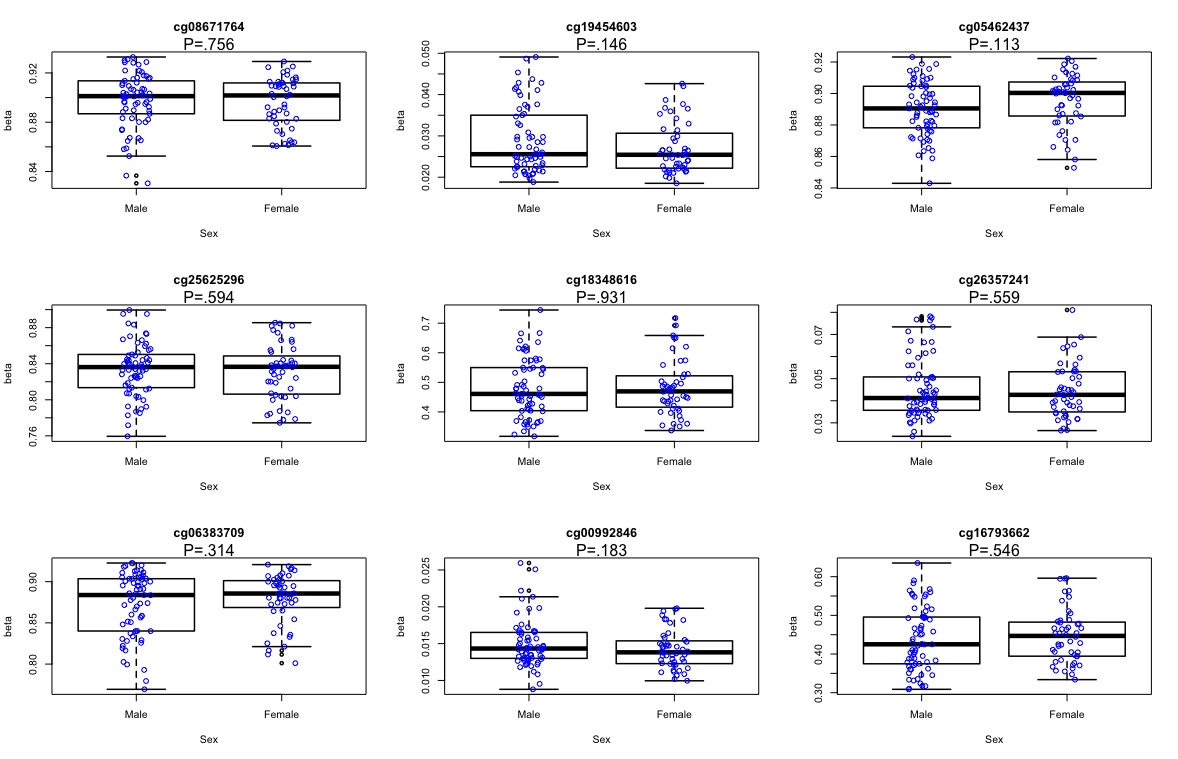


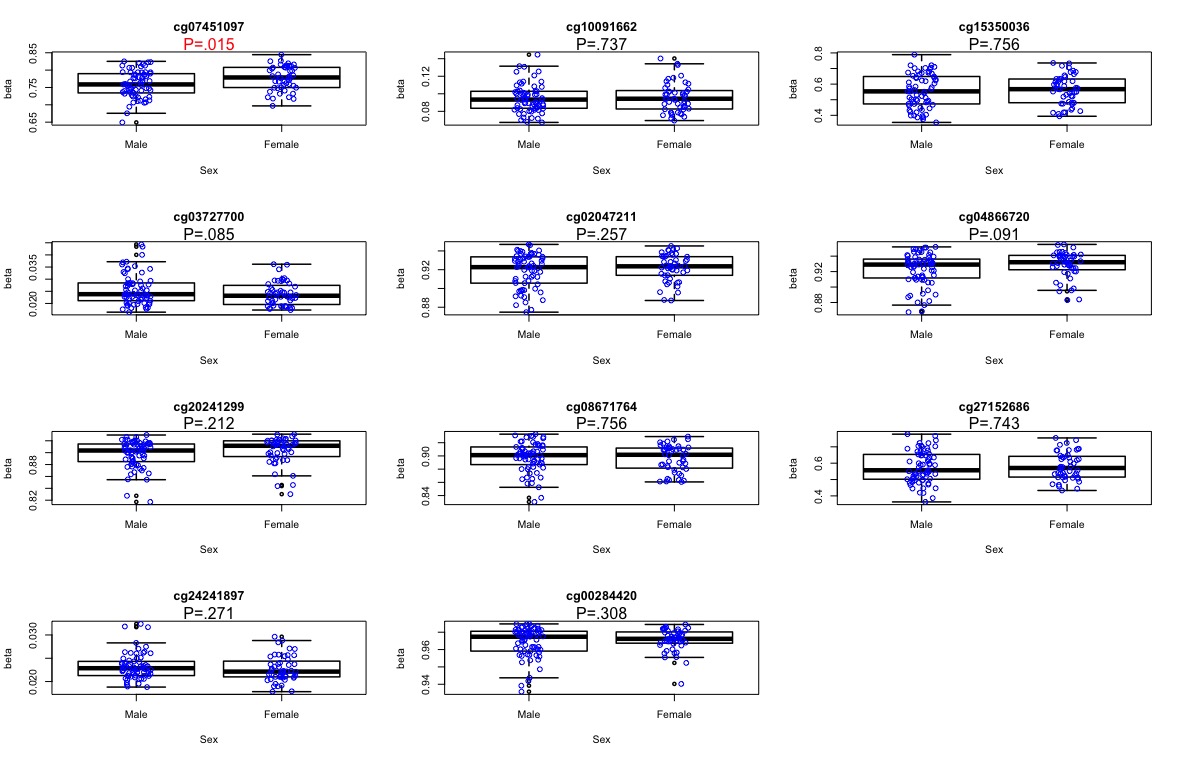


**Fig. S2.** Box plot of sex-stratified differences in DNA methylation β-values between baseline and follow-up for the CpG site significantly associated with neglect over follow-up in longitudinal epigenome-wide analyses.


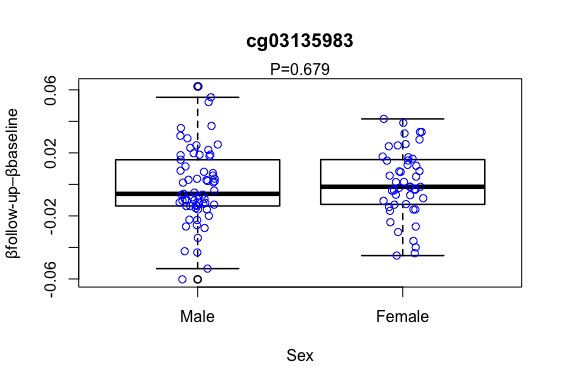

Supplement: Supplementary file 1 — Additional file 1. Supplemental Methods, Tables, and Figures. [file 13148_2022_1269_MOESM1_ESM.docx]
